# Effects of brief cognitive behavioral therapy on mental health in substance-related disorder: a randomized controlled trial

**DOI:** 10.1186/s12888-023-05413-4

**Published:** 2023-12-08

**Authors:** Seyed Mohammad Amin Alavi, Reza Davasaz Irani, Payam Fattahi, Sirus Pakseresht

**Affiliations:** 1 Faculty of medicine, Ahvaz Jundishapur University of Medical Sciences, Golestan Blvd, Ahvaz, 6135715794 Khuzestan Iran; 2 Ahvaz Jundishapur University of Medical Sciences, Ahvaz, Iran; 3 Medical Student, Shahid Beheshti University of Medical Sciences, Tehran, Iran; 4 Department of Psychiatry, Ahvaz Jundishapur University of Medical Sciences, Ahvaz, Iran

**Keywords:** Cognitive-behavioral therapy, Substance-related disorders, Mental Health, Anxiety, Insomnia, Psychotic disorders

## Abstract

**Background & objectives:**

Population and aging are major contributing factors influencing the increase in substance use disorder (SUD), which in itself affects mental health, particularly anxiety and depression. Cognitive behavioral therapy (CBT) and pharmacotherapy co-treatment are considered the gold standard for the treatment of SUD. Thus, the present study has been carried out to investigate the efficacy of brief CBT on the general health of opioid users.

**Methods:**

A randomized controlled trial (RCT) was conducted with forty opioid users whose addiction was dully confirmed by a psychiatrist at the drop-in center of the Ahvaz Jundishapur University of Medical Sciences. The patients were then randomly divided into two equal groups (*n* = 20). The control group was treated solely using methadone maintenance therapy (MMT); however, the intervention group underwent four sessions of CBT in addition to MMT. The general health questionnaire (GHQ) consisting of 28 items (Goldberg, 1979) was applied to both groups at the beginning and end of the study. The collected data was analyzed using IBM SPSS ver. 26, and data analysis was carried out using chi-square, t-test, Mann-Whitney, and Poisson regression model. P < 0.05 was statistically significant for all the aforementioned tests.

**Results:**

The mean age for the control and intervention groups were 37.95 ± 7.64 and 43.85 ± 9.92, respectively (*p* = 0.042). There was no statistically significant difference in terms of gender and levels of education (*p* = 0.311 and *p* = 0.540). Both groups differed statistically regarding marital status and occupation (*p* = 0.025 and 0.002). There was no significant statistical difference in all subclasses and the total scores of GHQ-28 for both groups, except for anxiety and insomnia in the intervention group (*p* = 0.038). After applying a Likert scale with a 23-point cut-off score, there was no statistically significant difference in terms of psychosis after intervention in the intervention group (*p* = 0.077).

**Conclusion:**

The results of the current study show that brief CBT is effective on psychiatric health, especially anxiety and sleep disorders, whereas brief CBT fails to affect the patient’s depression, somatic symptoms, and social dysfunction.

**Trial registration:**

The Iranian Registry of Clinical Trials (IRCT) approved the study design (IRCT registration number: IRCT20190929044917N1, registration date: 13/01/2020).

## Background

Substance Use Disorder (SUD) is among the leading healthcare challenges on a global scale [1]. The International Classification for Disease-11th Revision (ICD-11) by the World Health Organization (WHO) defines opioid dependency as a condition of opioid use regulation arising from recurrent or chronic opioid usage, which is characterized by the patient’s diminished ability to regulate usage, compounded by a growing preference for opioid use over other activities, and continued opioid use despite damage to health or subsequent adverse effects [2]. In North America, the term opioid use disorder, as per the definition in the Diagnostic and Statistical Manual of Mental Disorders 5 (DSM-5) published by the American Psychiatric Association, is commonly referred to as opioid dependency [3]. Since 1990, as a result of population growth and aging, the prevalence of opioid use disorders and age-standardized prevalence of opioid use disorders have increased significantly throughout the world in as such that in 2016, the prevalence of opioid use was 26.8 million cases [4]. Despite anti-SUD programs and policies, the incidence rate of SUD has yet to decrease. Among the various existing narcotic substances, opioid drugs are the most prevalent, severely causing global concern as stated in the global burden of disease (GBD) program report, which indicates that South Asia has one of the most concerning statistical spikes in terms of opioid use [1]. Illicitly manufactured heroin has been the predominant opioid used for non-medical purposes in most countries, except source nations and their bordering neighbors, as best typified by Afghanistan and Iran, where traditionally raw opium usage is a concern, albeit, in recent years, heroin injection has been on the rise in the aforementioned countries [5].

While mental health issues are significant comorbidities among non-medical prescription opioid users [6], anxiety and depression are the most commonly observed mental health problems in opioid use disorder [7].

Cognitive behavioral therapy (CBT) has proved to be an effective solution to a wide range of mental health disorders [8]. This could be attributed to the fact that CBT is a time-limited, multisession strategy that targets cognitive, affective, and environmental risks for substance abuse and teaches behavioral self-control skills to assist a person to achieve and maintain abstinence or reduce the risk of harm [9]. Moreover, CBT can significantly increase the self-esteem and self-efficacy of patients, improve coping skills, and enhance the individuals’ learning of behavioral skills to avoid substance abuse [10–12]. Previous studies revealed that CBT significantly reduces substance use temptations, the patients’ capability to take responsibility for the consequences of such temptations, and the quantity and frequency of opiate consumption [9, 13].

Brief CBT refers to reducing CBT content and condensing the standard 12–20 session duration into a shorter period of four to eight sessions focusing on a specific patient’s problem. It is believed that time-limited therapy might provide an added impetus for patients and therapists to operate with heightened efficiency and efficacy [14]. Previous studies highlighted the efficacy of brief CBT in different disorders, including anxiety, depression, eating disorders, and schizophrenia [15–18]. Brief CBT can also aid in alleviating symptoms, such as chronic pain, and has been found to be effective in reducing substance use and mitigating associated negative impacts [19–21]. International guidelines suggest opioid substitution treatment with long-acting opioids (methadone or buprenorphine) as the first-line pharmacological therapy for opioid dependency [22]. While CBT is the first-line treatment for SUD, pharmacotherapy, and CBT co-treatment are proposed as a gold standard for the treatment of SUD [23].

## Methods

### Aim

Prior studies mainly concentrate on standardized CBT for mental disorders and SUD; however, a longer duration of therapy may result in non-cooperation of the patients; therefore, the present investigation was carried out to examine the efficacy of brief CBT within an extremely condensed time frame (four sessions) on opioid users’ general health. To the best of our knowledge, there has not been any prior study so comprehensively investigating brief CBT.

### Study population

Opioid users were identified by a psychiatrist at the Ahvaz drop-in center, affiliated with Ahvaz Jundishapur University of Medical Sciences in January 2020, and a randomized controlled trial (RCT) was conducted with all of the opioid users who were registered and had medical records in the center. The inclusion criteria used in the study were (a) addiction to opioids based on ICD-11, (b) a period of methadone maintenance therapy (MMT), and (c) no prior history of CBT. The exclusion criteria were (a) having a medical or psychological disorder that explained the symptoms, (b) a concomitant medical or psychiatric disorder that required treatment or hospitalization, (c) inability to provide informed consent, and (d) leaving the center before the completion of CBT treatment. For the current study, forty opioid users were randomly divided into two equal groups using a table of random numbers (*n* = 20).

### Sample size

Since a sample size of 30 is recommended for the initial investigations [24], the study sample included 40 opioid users selected using a purposive sampling method and who subsequently received counseling at the Ahvaz drop-in center, affiliated with Ahvaz Jundishapur University of Medical Sciences.

### Randomization

Following the patient’s informed consent and the completion of the initial evaluation, the study coordinator randomly assigned participants to one of the two groups using a table of random numbers.

### The control group

Individuals in the control group received the same care and support they were receiving prior to starting the trial, including visits to their primary care physician, consecutive psychiatric evaluation sessions, and regular meetings with their care coordinator. All participants in the control group were treated using MMT.

### The intervention group

Individuals in the intervention group received the same care and support they were receiving prior to starting the trial, including visits to their primary care physician, consecutive psychiatric evaluations, and regular meetings with their care coordinator. However, individuals in the intervention group had four weekly sessions of CBT with a trained psychologist in addition to MMT. Each session was approximately 90 min; the summary is presented in Table 1.

**Table 1:** Cognitive behavioral therapy (CBT) summary

First Session: Motivational Interviewing	Motivation for change
	Strengthening commitment
	Monitoring self-behavioral
Second Session: Dealing with temptation and lapse	Dealing with temptation: an introduction
	What is temptation
	Methods to deal with temptation
	Preliminary plan for counter-acting temptation
	Methods to deal with lapses
Third Session: Regulation thoughts regarding substance abuse	Relation between thoughts and behaviors
	Triggers
	Discussing unrelated decisions
	Planning for festive and cheerful activities and events
Fourth Session: Relapse prevention	How to reject substance use
	Relapse prevention techniques

### Assessment

The general health questionnaire (GHQ) is a general screening test developed to assess the possibility of nonpsychotic mental disorders in primary care. A shorter version of this test consisting of 28 items was named GHQ-28. The specificity and sensitivity of this test were reported to be more than 80%. The GHQ-28 consists of four subclasses: somatic symptoms, anxiety and insomnia, social dysfunction, and severe depression [25]. The advantages of this test are conciseness (only five minutes for completion), adaptability to different languages, and psychometric features [26]. The results of the previous studies revealed that the Persian translation of the GHQ had favorable structural characteristics, establishing its reliability and validity as a suitable tool for assessing the psychological well-being of Iranian patients [27, 28].

GHQ-28 was utilized for both groups before the trial and after CBT in the intervention group. GHQ-28 requires individuals to discuss their general health over a four-week period using behavioral items with a 4-point (0, 1, 2, 3) Likert scale based on frequencies of experience defined as “not at all”, “no more than usual”, “rather more than usual” and “much more than usual” which in itself parallels the original scoring method [25, 29]. The minimum score observed was 0, and the maximum was 84. A higher score suggests higher levels of distress. Individuals with total scores ranging from 0 to 23 are categorized as non-psychiatric, whereas those with scores greater than 24 are classified as psychiatric. However, it is essential to note that this value should not be considered an absolute threshold; hence, each researcher is cautioned to establish a threshold score by utilizing the average value of their specific sample [30].

### Ethics approval

The ethics committee of Ahvaz Jundishapur University of Medical Sciences approved the study design (Ethics code: IR.AJUMS.HGOLESTAN.REC.1398.025). Informed consent was obtained from all subjects and/or their legal guardian(s) for participation in the study. All methods were carried out in accordance with the Declaration of Helsinki.

### Clinical trial registry

The Iranian Registry of Clinical Trials (IRCT) approved the study design (IRCT registration number: IRCT20190929044917N1, registration date: 13/01/2020).

### Statistical analysis

All patients were anonymized and given identification codes. Statistical analysis was carried out using IBM SPSS ver. 26 (IBM Corp., Armonk, NY, USA). The mean and standard deviation (SD) for continuous variables and the number and percentage for categorical variables were dully obtained. A Chi-square test was used to compare the categorical variables, and the Kolmogorov-Smirnov test was applied to evaluate whether the continuous variables were normally distributed. Parametric tests, such as the independent T-test, were used to compare the variables with normal distribution. Non-parametric tests, such as the Mann-Whitney U test, were further utilized to compare variables that did not have a normal distribution. In addition, a Poisson regression model was used to compare social dysfunction differences between the control and intervention groups after adjusting the effect of potential confounders such as occupation and marital status. *P* < 0.05 was considered as statistically significant.

## Results

The forty opioid users were divided into two groups: (a) the control group (*n* = 20) and (b) the intervention group (*n* = 20). The mean age for the control group was 37.95 ± 7.64, while the mean age for the intervention group was 43.85 ± 9.92. There was a statistical difference between groups regarding age (*p* = 0.042). Demographic data is summarized in Table 2. There was no significant statistical difference in terms of gender and levels of education. However, both groups were statistically different regarding marital status and occupation.

**Table 2:** Demographic characteristics

Demographic data		Control group Number (percent)	Intervention group Number (percent)	*p*
Gender	Male	20 (100)	19 (95)	0.311
	Female	0	1 (5)	
Marital status	Single	5 (25)	12 (60)	0.025
	Married	15 (60)	8 (40)	
Levels of education	High school	14 (70)	12 (60)	0.540
	Diploma	6 (30)	7 (35)	
	Bachelor’s degree	0	1 (5)	
Occupation	Unemployed	5 (25)	14 (70)	0.002
	Employee	15 (75)	4 (20)	
	Retired	0	2 (10)	

There was no statistically significant difference among both groups in terms of all subclasses of GHQ-28 and the total score before the intervention, except in terms of social dysfunction. However, the researchers observed a statistically significant difference in social dysfunction, anxiety, and insomnia, in addition to a difference in the GHQ-28 total score between the control and intervention groups after brief CBT. The results are shown in Table 3.

**Table 3:** GHQ-28 scores and difference between groups

Timepoint	Subclasses	Control group	Intervention group	*p*
Before CBT	Somatic symptoms	10.05 ± 3.804	9.2 ± 4.25	0.509
	Anxiety and insomnia	10.7 ± 3.511	10 ± 4.104	0.566
	Social dysfunction	9.85 ± 3.313	7.4 ± 3.185	0.022
	Severe depression	8.4 ± 3.676	8.4 ± 4.773	1.000
	Total score	39 ± 9.375	35 ± 10.026	0.200
After CBT	Somatic symptoms	9.85 ± 3.031	9.15 ± 4.120	0.544
	Anxiety and insomnia	11.6 ± 3.575	7.15 ± 4.043	0.001
	Social dysfunction	9.95 ± 3.379	6.3 ± 1.559	0.000
	Severe depression	8.1 ± 4.447	6.5 ± 3.547	0.216
	Total score	39.5 ± 10.216	29.1 ± 10.213	0.003

CBT: Cognitive Behavioral Therapy

Due to the statistically significant differences among both groups in terms of occupation, marital status, and social dysfunction, the authors utilized the Poisson regression model. After adjusting the effect of occupation and marital status, the multiple Poisson regression model results revealed no statistically significant difference (*p* < 0.05) for the mean value of social dysfunction among the control and intervention groups before CBT (Table 4).

**Table 4:** The Poisson regression model for adjusting the effect of occupation and marital status on social dysfunction among groups before the intervention

Variable	B	S.E.*	Wald	df	*P*-value	Exp(B)	95% CI for EXP(B)	
							Lower	Upper
Groups	0.198	0.134	2.181	1.000	0.140	1.219	0.937	1.586
Marital Status	-0.160	0.141	1.273	1.000	0.259	0.852	0.646	1.125
Occupation (Unemployed)	-0.203	0.269	0.569	1.000	0.450	0.816	0.481	1.384
Occupation (Employed)	-0.101	0.266	0.144	1.000	0.704	0.904	0.536	1.525

* Standard Error

Table 5 shows the results of the comparison between each group at the beginning and the end of the survey. The GHQ-28 results showed no significant difference in overall subclasses and total scores in both time intervals. However, the level of anxiety and insomnia showed a statistically significant decline in the intervention group after four sessions of CBT. Other subclasses and the total score showed no statistically significant difference in the intervention group before and after CBT.

**Table 5:** GHQ-28 scores and difference before and after intervention

Group	Subclasses	Before CBT	After CBT	*p*
Control group	Somatic symptoms	10.05 ± 3.804	9.85 ± 3.031	0.813
	Anxiety and insomnia	10.7 ± 3.511	11.6 ± 3.575	0.340
	Social dysfunction	9.85 ± 3.313	9.95 ± 3.379	0.909
	Severe depression	8.4 ± 3.676	8.1 ± 4.447	0.676
	Total score	39 ± 9.375	39.5 ± 10.216	0.761
Intervention group	Somatic symptoms	9.2 ± 4.25	9.15 ± 4.12	0.964
	Anxiety and insomnia	10 ± 4.104	7.15 ± 4.043	0.038
	Social dysfunction	7.4 ± 3.185	6.3 ± 1.559	0.151
	Severe depression	8.4 ± 4.773	6.5 ± 3.547	0.139
	Total score	35 ± 10.026	29.1 ± 10.213	0.052

CBT: Cognitive Behavioral Therapy

The researchers applied a Likert scale for scoring, in which the cut-off score was determined to be 23. There was no statistically significant difference in terms of psychotic and non-psychotic opioid users in the intervention group before and after CBT (Table 6). However, the control and intervention groups statistically differed after CBT.

**Table 6:** Likert scoring with 23 points cut off

Timepoint		Control group	Intervention group	*p*
Before intervention	Non-psychotic	2 (10)	3 (15)	0.633
	Psychotic	18 (90)	17 (85)	
After intervention	Non-psychotic	2 (10)	8 (40)	0.028
	Psychotic	18 (90)	12 (60)	
		1.000	0.077	

## Discussion

A significant effect of SUD, which the current researchers focused on, is its impact on psychiatric comorbidities, specifically anxiety and insomnia. Treating individual patients using novel SUD management techniques effectively decreases the susceptibility of society to opiate abuse as a whole and can be utilized as a preventative measure. In recent decades, several clinical trials using traditional treatment methods were carried out, yet they were proven to be ineffective; by contrast, CBT has proven effective in treating various mental disorders, including substance abuse. In the current study, the researchers examined the efficiency of brief CBT on mental disorders in SUD and concluded that the application of the treatment is effective on insomnia and anxiety among opiate users.

The results in Table 3 display the GHQ_28 total score difference between the intervention and control groups. While GHQ-28 scores showed no statistically significant difference among both groups during the study period, the scores for anxiety and insomnia among the intervention group decreased significantly after CBT. The results are summarized in Table 5. It was observed that the difference between the total scores before and after CBT in the intervention group was not statistically significant (*p* = 0.052), possibly due to the small sample population. According to Table 6, the number of psychotic patients did not show any significant statistical difference among both groups before the intervention. However, after four sessions of CBT, the psychotic patients of the intervention group declined (insignificantly, at a p-value of 0.077, which can also be a result of the small sample size); this in itself resulted in a significant difference among both two groups. An essential aspect of the study was CBT’s effect on psychiatric comorbidities of SUD. The researchers found that patients were significantly less morbid after four sessions of CBT, which could be attributed to the primary effect of CBT and chiefly associated with the effect of CBT on reducing the consumption of opiates.

Sitnikova et al. (2019) studied the effectiveness of six sessions of CBT on patients with undifferentiated somatoform disorder. The study found no difference between CBT and standard care regarding the severity of somatic symptoms [31], which in itself was similar to the current study’s findings. Liu et al. (2019) performed a meta-analysis to investigate the effect of CBT on medically unexplained physical and somatoform symptoms. The results show that CBT can significantly reduce anxiety, depression, and social function [32]. The results of Liu’s study contradicted the findings of the current study (except for anxiety), which could be related to substance withdrawal since other groups of psychological patients were not included in the study.

Zhang et al. (2016) investigated the efficacy of 12 weeks of group CBT among patients with mild depression and found improvement in terms of social functioning, which was in contrast with the current study’s findings [33]. The reason for the discrepancy might be due to different studied illnesses and the duration of CBT.

Kamarzarin et al. (2019) investigated SUD patients and found that depressive behaviors after CBT significantly improved; however, in the present study, such an effect was not observed [34]. The differences between brief and long CBT interventions for depression might explain the variation in depression symptoms in the abovementioned study and the current study. In another study by Bador and Kerekes (2020), the effect of integrated intensive CBT (five days a week within a four-month period) during addiction care was evaluated and revealed that an improvement in the extent of depression among the studied patients occured [35]. The results of Bador’s study directly contrasted the current study’s findings, which might be due to the intensity and duration of the CBT.

Speed et al. (2022) carried out a randomized controlled trial among 21 patients with substance use disorder to compare the efficacy of eight sessions of group-based CBT with nine hours of behavioral health sessions on insomnia. It was observed that insomnia symptoms were reduced in both groups over time; however, the decline rate in the CBT group was higher as compared to the control group (80% versus 25%) [36]. The results were in line with the current study. A pilot investigation was further conducted among veterans identified with cannabis use disorder, whereby it was observed that the utilization of the CBT Coach mobile application (two-week intervention) had positive outcomes in terms of diminishing cannabis consumption and enhancing sleep quality [37], which was in line with the current study.

CBT is also considered to be an effective first-line treatment option for anxiety [38]. Cully et al. (2017) investigated medically ill veterans to study the efficacy of eight sessions of brief CBT. The study revealed that brief CBT improved the symptoms of anxiety among the studied patients [39], which was in line with the current study’s findings. Golshani et al. (2020) carried out a systematic review and meta-analysis of CBT psychiatric effects on Infertile Iranian females before and after CBT. The results prove CBT’s positive effect on anxiety [40], which was in itself aligned with the current study’s findings.

The effect of brief CBT has yet to be investigated extensively. Roos et al. (2020) performed a review study of clinical trials to differentiate CBT’s short-term and long-term effects on SUD patients and evaluate computer-based CBT on brief or long-term outcomes. The results showed that long-term effects on coping skills were more significant than short-term outcomes [41]; hence, the results of the current study can be discussed in light of CBT’s effect on patients’ awareness of themselves and their situation, in addition to an increase in self-esteem and self-efficacy.

## Conclusion

The results of the current study show that brief CBT is effective on psychiatric health, especially anxiety and sleep disorders, but not on patients’ depression, somatic symptoms, and social dysfunction. The researchers believe that in the future, brief CBT could aid psychiatrists and psychologists to control anxiety and sleep disorders. The short timeframe of the intervention used in the current study can increase the cooperation of the SUD patients; moreover, controlling anxiety by implementing brief CBT for SUD patients can improve their quality of life.

## Limitations

The main limitation imposed on the current investigation was the low study population. The researchers highly recommend studying CBT efficacy in SUD among larger populations. It is also recommended that future studies focus on the differences between brief and long-term CBT in SUD. The present study has not determined the efficacy of brief CBT on patients’ consumption and recurrence. Another limitation of the current study is the short-term follow-up duration, for which the authors highly recommend a longer follow-up duration for future studies; moreover, computerized CBT should be the pivot for further studies, and these studies should include more female patients in order to investigate the efficacy of brief CBT among women.

## Data Availability

The data supporting this study’s findings are available upon reasonable request from the corresponding author.
